# Effects of Barefoot and Shod Conditions on the Kinematics and Kinetics of the Lower Extremities in Alternating Jump Rope Skipping—A One-Dimensional Statistical Parameter Mapping Study

**DOI:** 10.3390/bioengineering10101154

**Published:** 2023-10-02

**Authors:** Jun Li, Kaicheng Wu, Dongqiang Ye, Liqin Deng, Jichao Wang, Weijie Fu

**Affiliations:** 1 School of Exercise and Health, Shanghai University of Sport, Shanghai 200438, China; 2111516014@sus.edu.cn (J.L.); 2221517012@sus.edu.cn (J.W.); 2 School of Health and Nursing, Wuxi Taihu University, Wuxi 214000, China; 3 Shanghai Warrior Shoes Co., Ltd., Shanghai 200082, China; ydq@warriorshoes.com

**Keywords:** rope jumping, shoe conditions, statistical parameter mapping, lower extremity

## Abstract

Purpose: To explore the difference in the biomechanics of the lower extremity during alternating jump rope skipping (AJRS) under barefoot and shod conditions. Methods: Fourteen experienced AJRS participants were randomly assigned to wear jump rope shoes or be barefoot (BF) during the AJRS at a self-selected speed. The Qualisys motion capture system and Kistler force platform were used to synchronously collect the ground reaction forces and trajectory data of the hip, knee, ankle, and metatarsophalangeal (MTP) joints. One-dimensional statistical parameter mapping was used to analyze the kinematics and kinetics of the lower extremity under both conditions using paired t-tests. Results: Wearing shoes resulted in a significant decrease in the ROM (*p* < 0.001) and peak angular velocity (*p* < 0.001) of the MTP joint during the landing phase. In addition, the MTP joint power (*p* < 0.001) was significantly larger under shod condition at 92–100% of the landing phase. Moreover, wearing shoes reduced the peak loading rate (*p* = 0.002). Conclusion: The findings suggest that wearing shoes during AJRS could provide better propulsion during push-off by increasing the MTP plantarflexion joint power. In addition, our results emphasize the significance of the ankle and MTP joint by controlling the ankle and MTP joint angle.

## 1. Introduction

Jump rope is a popular high-intensity aerobic exercise among sports enthusiasts due to its convenient spatial requirements and ease of execution [1,2]. The activity has gained significant global recognition, with over 50 countries boasting professional jump rope clubs [3]. Jump rope is a highly favored physical activity among adolescents, as evidenced by the annual participation of approximately 1 million students from 4000 schools in Canada [4]. Moreover, numerous educational institutions in Asia integrate rope skipping into their students’ athletic endeavors [5]. Jump rope skipping is even incorporated into the Middle School Entrance Examination of physical education in China [6].

Jump rope has gained popularity among athletes and school-aged students. As such, enhancing jump rope performance has emerged as a focus for both athletes and school-aged students. Biomechanists have been studying ways to optimize sports equipment to improve athletic outcomes. This has led to a growing interest in understanding the biomechanics of jump rope movements and identifying factors that can positively impact performance [1,7,8]. There are various types of rope skipping, but they all involve continuous vertical jumps at a relatively low height. The most common technique used in rope skipping is known as alternating jump rope skipping (AJRS). The AJRS refers to jumping with one foot at a time and landing with the other foot alternately. For instance, one would jump with the left foot and land with the right foot and then switch to jumping with the right foot and landing with the left foot [9]. In addition, it is a complex cyclic movement that necessitates the synchronization of the upper and lower extremities to accomplish the rotation of the rope. It entails the consecutive landing of the left and right feet with a minimal jumping height and requires the lower extremity muscle to generate propulsion. The lateral edge of the forefoot and midfoot serves as the primary impact region during AJRS [7], with the ankle joint demonstrating the most joint power and range of motion (ROM) [8] among the lower extremity joints. AJRS had a greater hip flexion angle than bounced jump in the sagittal plane and a smaller hip adduction angle in the frontal plane [9]. It also had a greater GRF and loading rate than bounced jump [2,9]. Upon the foot making contact with the ground at the end of the take-off stage, the lower extremity initiates a response by adapting the stiffness of the lower extremity. This involves the adaptation of muscles of the lower extremity [9].

In addition to the triceps surae muscles, the plantar flexor muscles positioned at the rear of the ankle joint are also essential for propulsion, contracting at the metatarsophalangeal (MTP) joint to generate propulsion during jumping and running [9,10,11]. Taking a training perspective, coaches emphasize the importance of training the ROM of the MTP joint and the strength of the toe flexors in jumping events [12,13]. Such coaches posited that stabilizing the MTP joint through targeted exercises can increase the strength output of adjacent joints and strengthen the control of the MTP joint angle, leading to improved athletic performance. The contribution of the MTP joint in AJRS is thus crucial.

Efficient execution of the AJRS demands optimal interplay among the lower extremity joints and the integration of the lower extremity as a functional entity [14]. As a medium of the foot and ground, footwear has been shown to have the potential to enhance athletic performance [15,16,17]. Footwear designed with a higher stiffness has been observed to alter the transmission ratio of the ankle joint during running, which in turn can enhance running performance [18,19]. Similarly, Yu et al. [1,7] found that jump rope shoes with a low degree of toe uplift and a thin sole could significantly affect jump rope performance. Footwear that reduces plantar pressure may be more beneficial for long-term jump rope exercise [8].

Barefoot (BF) exercise, which represents a special footwear condition, has been recognized as a fundamental basis for exploring specialized footwear, particularly because BF exercise denotes an unimpeded state. Comparing the effects of BF exercise with those of shod exercise can aid in understanding how footwear impacts movement [20]. Srs et al. reported that some athletes choose to perform rope skipping under barefoot conditions to strengthen the stabilizer muscles in their feet and ankles, which leads to improved stability and balance [21,22]. However, the potential difference in AJRS under shod and BF conditions, and whether such differences may enhance athletic performance, remain inconclusive. In addition, there remains a paucity of research on the biomechanics of the “foot–shoe complex” in AJRS [23], and a consensus on whether discrepancies in lower extremity joint biomechanics exist between jump rope with and without shoes, and if the latter can promote performance, has yet to be established.

Therefore, this study aimed to examine the effect of footwear on the biomechanics of the lower extremity joints—specifically, the ankle and MTP joints—and determine whether it can improve AJRS performance. We hypothesized that compared with barefoot conditions, wearing shoes would result in reduced ground contact and flight times during AJRS, decreased ROM and peak angular velocity of the ankle and MTP joints, and increased ankle and MTP joint power.

## 2. Methods

### 2.1. Participants

Fifteen experienced male AJRS participants were recruited for this study [24] (Table 1). The inclusion criteria required that the participants possess more than three years of trained experience, with the ability to execute five consecutive AJRS and perform more than 140 jumps per minute. Furthermore, all participants were asked to test with a dominant leg, defined as the preferred kicking leg [25], and to not have experienced any lower extremity injuries or related illnesses within the past six months and not engage in strenuous exercise for 24 h before the study. Before the experim ents, all participants were required to provide informed consent and signed an informed consent form. The study was approved by the Institutional Review Board of Shanghai University of Sports (No. 102772023RT029).

### 2.2. Instrumentation

**Figure 1:**
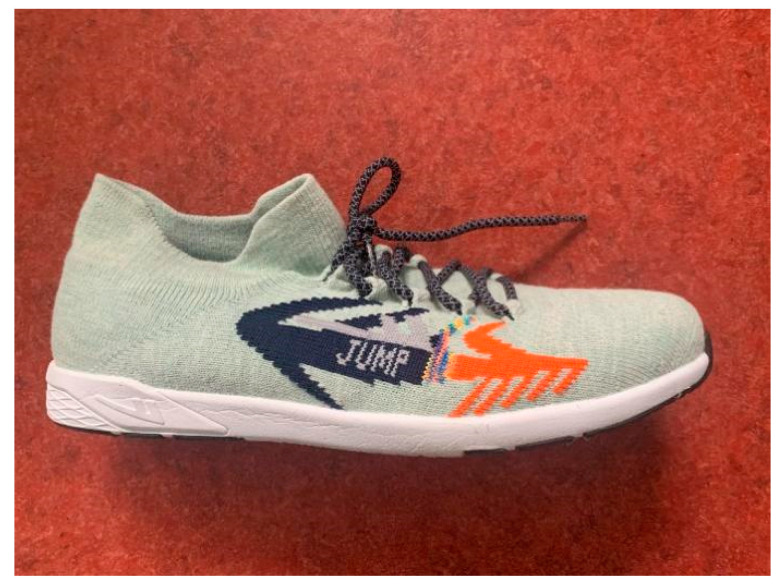
Experimental footwear.

An eight-camera motion capture system (Qualisys Motion Capture System^®^ (QUALISYS MEDICAL AB, Gothenburg, Sweden)) was used to collect kinematic data at a sampling rate of 200 Hz. Ground reaction force (GRF) data were measured using one 90 cm × 60 cm × 10 cm force platform (Kistler, Type 9287B, Switzerland) at a sampling rate of 1000 Hz. The kinematics and GRF data were simultaneously collected using the Qualisys system. Jump rope shoes (39 mm toe height, 7 mm heel-to-toe drop, 1.8 N·m shoe stiffness, 192 g, China) were used as experimental shoes (Figure 1).

### 2.3. Procedures

**Figure 2:**
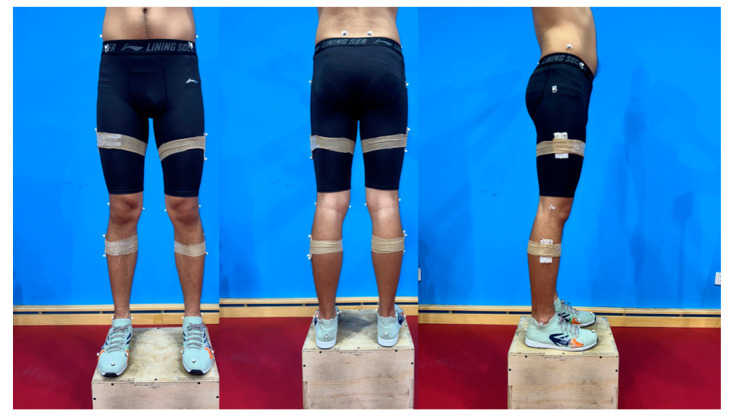
Marker set used in this study.

**Figure 3:**
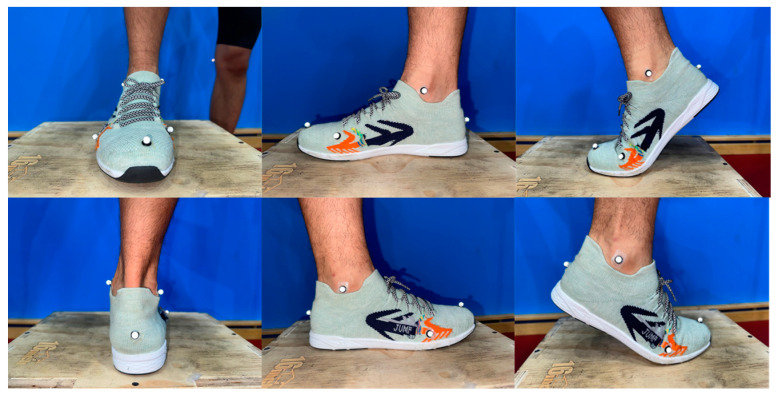
Marker set of the ankle and MTP joints under shod conditions.

**Figure 4:**
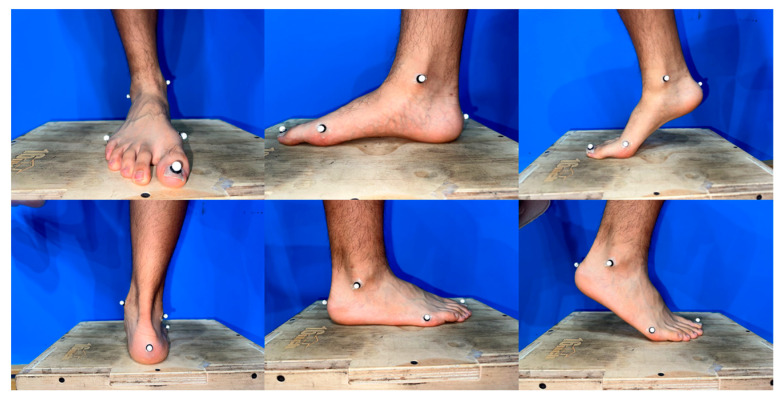
Marker set of the MTP joints under barefoot conditions.

Before conducting the formal experiment, the laboratory personnel conducted a pre-experiment. All members of the experimental team were well acquainted with the experimental procedure, standard operating protocols, and equipment calibration. They also ensured the availability of experimental supplies and set up the experimental site. At the beginning of the experiment, participants were provided with uniform clothing, including a vest, shorts, and socks. Following a 30 s warm-up consisting of rope skipping under both conditions, the participants wore tight shorts and had reflective markers affixed to designated locations on their lower extremities [26,27]. To define the hip, knee, ankle, and MTP joints, 36 infrared retroreflective markers were bilaterally attached [9,28]. The anatomical locations of the markers were the right/left ilium crest tubercle, right/left posterior superior iliac spine, right/left femur greater trochanter, right/left anterior superior iliac spine, right/left femur lateral epicondyle, right/left femur medial epicondyle, right/left fibula apex of the lateral malleolus, right/left tibia apex of the medial malleolus, right/left head of the fifth metatarsals, right/left head of the first metatarsus, right/left posterior surface of the calcaneus, and right/left big toes [9]; the tracking markers were placed on the thigh and shank (Figure 2). The placement of markers was performed by an experienced researcher (5 years) majoring in Biomechanics. The MTP joint was considered as a single joint rotating about an axis oblique to the sagittal plane defined by the first and fifth metatarsal markers (Figure 3). The MTP angle was defined as the angle between the forefoot and rearfoot segments in relation to a standing calibration for a reference measurement in the sagittal plane [28] (Figure 4). After the collection of the static posture, the participants selected a self-selected speed, and the order of barefoot vs. shod skipping was randomized during the test to avoid learning or fatigue effects. If errors occurred during the test, adjustments were made to ensure complete and stable data collection for at least five consecutive stable trials within a 30 s AJRS process. If necessary, the test was repeated after a period of rest until the data collection requirements were met.

### 2.4. Data Processing

The 3D coordinates of the reflective markers were filtered through a Butterworth fourth-order, low-pass filter at a cut-off frequency of 7 Hz with V3D software (v3, C-Motion, Inc., Germantown, MD, USA) [29]. The GRF data were filtered at a cut-off frequency of 100 Hz [27,29]. The initial contact was defined as the time at which the GRF exceeded 50 N [3].

The joints selected for analysis were the ankle and MTP joints. The three-dimensional kinematics data of these joints and GRF data were processed and analyzed during the landing phase. Specifically, the selected performance variables include the center of mass displacement and ground contact time. The selected kinematic variables include the ROM of the ankle and MTP joints, the peak angular velocity of the ankle and MTP joint, and the ankle and MTP joint angle during the landing phase. The selected kinetic variables include the GRF, peak loading rate, lower extremity stiffness, joint moment, and joint power of the ankle and MTP. A reliability test was conducted in the pilot experiment using the intraclass correlation coefficient (ICC(3,1)) of multiple measurements taken by the same experimenter. The experimenter conducted two measurements on the same set of participants for the same variable [30,31]. The repeated measurements were conducted on different days (2.5 ± 1.3). The results showed the good reliability of the MTP angle collected and calculated using this model and measurement during AJRS (ICC = 0.9332). 

The following are the definitions and specific calculation methods of the selected variables (Table 2):

### 2.5. Statistics

SPSS 22.0 (SPSS Inc., Chicago, IL, USA) was used to complete the statistical analysis, and all data were presented as the mean ± standard deviation. Data normality was first examined with the Shapiro–Wilk test. Paired sample *t*-tests were used to compare the influence of barefoot and shod conditions on each parameter; otherwise, Wilcoxon signed-rank tests were used. One-dimensional statistical parameter mapping (SPM), a statistical analysis methodology (SPM1d version 0.4, https://www.spm1d.org, accessed on 23 September 2021) founded on time-space smoothing and standardized data testing, was used for the statistical analysis of the kinematics and kinetics of the lower extremity joints [34]. The 95% confidence intervals and effect size (Cohen’s *d*) of each parameter in this study were calculated. A Bonferroni correction was conducted to avoid type I errors for the paired sample *t*-test and Pearson correlation and adjust the significance level to 0.004. All analyses were performed using Matlab R2022a (Mathworks, Natick, MA, USA).

## 3. Results

### 3.1. Performance

Compared with the BF condition, the ground contact time and the center of mass displacement have no significant difference under shod conditions (Table 3).

### 3.2. Kinematics

**Figure 5:**
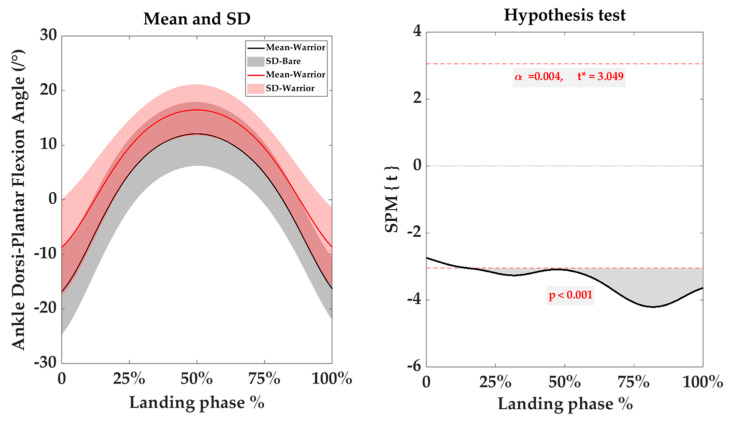
SPM analysis of the joint angle on the sagittal plane of the ankle (Mean, SD); BF in black, shod in red.

During the landing phase, compared with the BF condition, in the sagittal plane, the ROM of the ankle joint was similar under both conditions, while the ROM of the MTP was significantly decreased (*p* < 0.001) in the shod condition (Table 4). Interestingly, significant differences in the ankle dorsi-plantar flexion angle were observed in 10–100% of the landing phase (*p* < 0.001), with a greater dorsiflexion angle observed in the shod condition (Figure 5). Additionally, the ROM of the MTP joint was significantly decreased in the shod condition (*p* < 0.001) of the landing phase.

During the landing phase, in the sagittal plane, the peak angular velocities of MTP joints (*p* < 0.001) decreased in the shod condition (Table 4).

### 3.3. Kinetics

**Figure 6:**
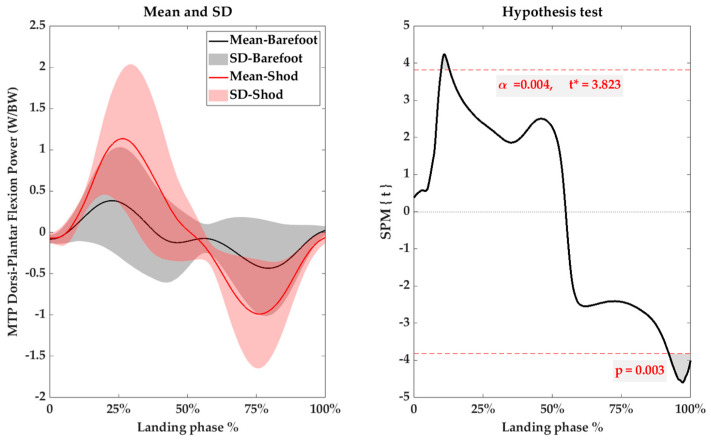
SPM analysis of the sagittal joint power of the MTP Dorsi-Plantar Flexion. SD is the standard deviation. Barefoot in black, shod in red.

Compared with the BF condition, the peak loading rate was significantly smaller under shod conditions (*p* = 0.002) (Table 5), whereas no significant differences were detected in the peak joint moment of the ankle and MTP joint. The ankle joint exhibited a high power output both in the BF and shod conditions, while the MTP joints had relatively smaller peak power outputs. But there were no significant differences in the peak power outputs of the ankle and MTP joint between the two conditions. The SPM analysis showed no significant differences in the landing phase of the joint power of the ankle, but the MTP Dorsi-Plantar Flexion Power was significantly smaller than that under shod conditions in the last 8% of the landing phase (Figure 6).

## 4. Discussion

The purpose of this study was to investigate the effects of BF and shod conditions on lower extremity joint biomechanics during AJRS. The results supported our hypothesis that the MTP dorsi-plantar flexion power was increased in the shod condition. However, there were no significant differences in the duration of the landing phase and the center of mass displacement. Previous studies found that the performance of skipping rope is influenced by various factors such as the ground contact time, flight time, center of mass displacement, and joint angles [35,36]. Jump rope athletes aim to complete as many jumps as possible within 30 s, and thus, the recommended strategy that controls the joint angle of the lower extremities during competition is to minimize the duration of the flight and landing phases in order to increase the number of jumps [35,36]. Although, in this study, it was observed that the ground contact time and center of mass displacement showed no significant differences under both conditions, significant differences were observed in the ankle and MTP joint angles and the power. In other words, they employed different jump rope strategies under both conditions, especially during push-off.

According to research findings, significant variations in spatiotemporal variables were observed in shod conditions as compared with BF conditions [20,37]. Generally, kinematic alternations predominantly manifest in the knee joint, ankle joint, and foot under shod conditions [37,38]. Athletes with shoes mainly adjust their jump rope strategy by modulating the joint angles of their lower limbs at touchdown. In this study, compared with the BF condition, shod AJRS led to a significant increase in the joint angle of the ankle joint. This finding may indicate the influence of footwear on the AJRS pattern of the ankle joint. The support and stability provided by the footwear could lead to adjustment in the ankle joint angle to accommodate the AJRS in shod conditions [17,39].

Compared with the BF condition, significant decreases were observed in the ROM of the MTP joint. Specifically, the ROM decreased by 26.6%. This change was consistent with previous reports in other sports [13,40,41] and was confirmed by SPM analysis. However, the change in the MTP joint during AJRS was greater than that in running, which might be attributed to the mechanics of AJRS, wherein AJRS required propulsion through the forefoot [27]. Therefore, the effect of wearing shoes on the dorsiflexion of the MTP joint was more pronounced. In addition, Kim et al. reported that training focused on controlling the ankle and MTP joint improved jump rope performance, with the skilled group demonstrating significantly less ankle dorsiflexion compared with the unskilled group [35].

Although no significant differences were found in the peak angular velocity of the ankle joint under both conditions (*p* = 0.082), the SPM results showed that the joint power of the MTP was significantly greater at push-off under BF conditions (Figure 6), which suggests that wearing shoes may enhance the joint’s ability to generate force during push-off. This finding implies that wearing shoes improves the control and force generation capacity, leading to more efficient utilization of joint power during AJRS, thus promoting better jump rope performance. Therefore, it could be proposed that wearing shoes can enhance the propulsion at the push-off [42,43]. This decrease may indicate that wearing shoes restricts the natural movement of the MTP joint, which could affect the coordination and flexibility of AJRS, potentially leading to decreased agility or restricted performance [13]. This highlights the importance of the MTP joint during AJRS and emphasizes the need for careful consideration when choosing footwear for jump rope activities, and the design of jump rope shoes should take into account the MTP joint. In addition, wearing shoes during AJRS could shift the forefoot propulsion point forward, extending the dynamic arm and increasing the longitudinal bending stiffness of the ankle joint [44,45,46]. This finding suggested a potential mechanism for improving AJRS performance.

According to previous studies, vertical GRF*_max_* is significantly reduced under BF conditions [47,48,49]. Although there was no significant difference in the GRF*_max_* and time series of GRF under both conditions, the observed results indicated a reduction of 28.5% in the peak loading rate under shod conditions (Table 5), which suggests that wearing shoes may provide a better buffer during AJRS, and this strategy was achieved by adjusting the angle of the lower extremity. It suggests that the interaction between the “foot–shoe complex” plays a significant role in AJRS, potentially influencing joint stability, shock absorption, and performance [50,51]. Yu et al. found that modern jump rope shoes utilize elastic materials to absorb impact, which showed a lower plantar pressure than other shoes during jump rope [1,7]. In theory, the stiffness of the lower extremity during exercise plays a crucial role in the storage and release of elastic energy, which can significantly impact the exercise technique and performance, particularly in activities involving jumping and running [17,52]. Efficient storage and recoiling elastic energy is believed to enhance the efficiency of movements, allowing athletes to achieve higher jumps and perform explosive actions more effectively. In this study, it was observed that there was no significant difference in lower extremity stiffness between the two conditions. These results suggest that the tested footwear, likely designed with specific shock-absorbing capabilities, may effectively dampen the impact forces generated during AJRS, leading to comparable lower extremity stiffness as compared to the barefoot condition [53].

This study has certain limitations. First, while the participants underwent a similar level of AJRS and were provided with an adaptation to BF skipping before the trial, it is important to consider that there may still be individual differences in lower extremity biomechanics. This is because all participants were inexperienced in barefoot AJRS, and they may not have fully adapted to the BF condition. In addition, this study focused exclusively on a widely acknowledged jump rope shoe that is commercially available. However, it is suggested that subsequent research endeavors aim to examine a diverse set of jump rope footwear, encompassing different levels of rigidity and variations in the differential between the toe and heel, or alternatively, they concentrate specifically on the malleability and reactivity of jump rope shoes to augment athletes’ foot flexibility and sensory perception [54]. Finally, the measurement of the MTP joint angle using the marker set of the MTP joints under shod conditions could not acquire the real skeletal motion.

## 5. Conclusions

Based on the variables examined in this study, it was observed that wearing shoes was insufficient to enhance AJRS performance compared to the BF condition, but it did alter the biomechanics of AJRS. Specifically, wearing shoes during AJRS increased the MTP plantarflexion joint power and ankle dorsi-plantar flexion angle and decreased the ROM and peak angle velocity of the MTP joint during the landing phase. These findings emphasize the significance of the ankle and MTP joint during AJRS and suggest the importance of controlling the ankle and MTP joint angles to provide better propulsion during push-off. 

## Figures and Tables

**Table 1:** The basic information of the participants (mean ± SD).

No.	Age (Years)	Height (cm)	Weight (kg)	Rope Skipping Level (n/min)
15	25.3 ± 2.9	72.3 ± 6.7	175.7 ± 3.8	140.6 ± 5.1

**Table 2:** The definitions or calculations of the selected variables.

No.	Variable	Definition or Calculation
1	Center of mass displacement	the movement or shift of the center of mass of the participant during AJRS
2	Ground contact time	the duration of time in which the feet make contact with the ground during each AJRS
3	Peak angular velocity	the maximum rotational speed or rate of change of the angular displacement at a joint during the landing phase of AJRS
4	ROM	the range of motion of the lower extremity joints during the landing phase
5	Peak loading rate	calculated as the peak rate of change of the ground reaction force (*GRF*) during the landing phase of AJRS [32]
6	Lower extremity stiffness (*k_leg_*)	lower extremity stiffness was calculated by taking the ratio of the leg vector projected ground reaction force (*GRF_proj_*) to the compression of the leg (*leg*_*comp*_) at the instant when the compression was maximal [32]: *k_leg_* = *GRF_proj_/leg_comp_*
7	Joint moment	calculated by inverse dynamics according to Hof [33,34]
8	Joint power	calculated as the dot product of the normalized joint moment and joint angular velocity

**Table 3:** Comparison of performance variables during the landing phase under the barefoot and shod conditions.

Variables	Shoe Conditions		*p*-Value	95% CI	Cohen’s d
	Barefoot	Shod			
Ground contact time (ms)	263 ± 40	239 ± 36	0.031	[6.59; 41.33]	0.62
Center of mass displacement (cm)	9.7 ± 1.5	8.7 ± 1.7	0.01	[0.10; 1.86]	0.76

Note: 95% CI = 95% confident interval.

**Table 4:** Comparison of kinematic variables under the barefoot and shod conditions.

Variables	Shoe Conditions		*p*-Value	95% CI	Cohen’s d
	Barefoot	Shod			
ROM*_ankle_* (°)	28.7 ± 4.1	27.1 ± 4.1	0.044	[0.05; 2.78]	0.57
ROM*_MTP_* (°)	27.2 ± 5.6	16.7 ± 4.4	<0.001 *	[6.74; 13.33]	0.89
ω*_ankle_max_* (°·s^−1^)	162.7 ± 20.2	145.3 ± 36.5	0.082	[−2.75; 37.54]	0.69
ω*_MTP_max_* (°·s^−1^)	98.7 ± 15.1	62.17 ± 23.0	<0.001 *	[22.56; 50.41]	0.86

Note: 95% CI = 95% confident interval; ROM*_ankle_* and ROM*_MTP_* are the ROM of the ankle and MTP joints, respectively, in the sagittal plane; ω*_ankle_max_* and ω*_MTP_max_* are the peak angular velocities of the ankle and MTP joint in the sagittal plane; ^*^ *p* < 0.004, which indicates significant differences in barefoot and shod conditions.

**Table 5:** Comparison of the kinetic variables during the landing phase under the barefoot and shod conditions.

Variables	Shoe Conditions		*p*-Value	95% CI	Cohen’s d
	Barefoot	Shod			
GRF*_max_* (BW)	2.8 ± 0.3	2.9 ± 0.3	0.237	[−0.26; 0.07]	0.237
Peak loading rate (BW/s)	62.0 ± 12.3	44.3 ± 13.9	0.002 *	[8.38; 31.07]	0.82
k*_leg_* (BW/m)	30.2 ± 6.5	35.4 ± 10.7	0.033	[−10.13; −0.49]	0.33
Peak ankle Dorsi-Plantar flexion moment (N·m/BW)	2.4 ± 0.8	2.8 ± 0.6	0.057	[−0.79; 0.05]	0.57
Peak MTP Dorsi-Plantar flexion moment (N·m/BW)	0.6 ± 0.4	0.5 ± 0.3	0.377	[−0.14; 0.24]	0.28
Peak ankle Dorsi-Plantar flexion power (W/BW)	9.1 ± 1.7	10.9 ± 2.7	0.049	[−3.64; −0.007]	0.69
Peak MTP Dorsi-Plantar flexion power (W/BW)	0.8 ± 0.7	0.7 ± 0.5	0.779	[−0.20; 0.26]	0.71

Note: GRF*_max_* is the peak GRF; BW is the body weight; * *p* < 0.004, which indicates significant differences in barefoot and shod conditions.

## Data Availability

The data are available upon request.
